# Early Warning of Cotton Bollworm Resistance Associated with Intensive Planting of Bt Cotton in China

**DOI:** 10.1371/journal.pone.0022874

**Published:** 2011-08-09

**Authors:** Haonan Zhang, Wei Yin, Jing Zhao, Lin Jin, Yihua Yang, Shuwen Wu, Bruce E. Tabashnik, Yidong Wu

**Affiliations:** 1 College of Plant Protection, Nanjing Agricultural University, Nanjing, China; 2 Key Laboratory of Integrated Management of Crop Diseases and Pests (Nanjing Agricultural University), Ministry of Education, Nanjing, China; 3 Department of Entomology, University of Arizona, Tucson, Arizona, United States of America; Ghent University, Belgium

## Abstract

Transgenic crops producing *Bacillus thuringiensis* (Bt) toxins kill some key insect pests, but evolution of resistance by pests can reduce their efficacy. The predominant strategy for delaying pest resistance to Bt crops requires refuges of non-Bt host plants to promote survival of susceptible pests. To delay pest resistance to transgenic cotton producing Bt toxin Cry1Ac, farmers in the United States and Australia planted refuges of non-Bt cotton, while farmers in China have relied on “natural” refuges of non-Bt host plants other than cotton. Here we report data from a 2010 survey showing field-evolved resistance to Cry1Ac of the major target pest, cotton bollworm (*Helicoverpa armigera*), in northern China. Laboratory bioassay results show that susceptibility to Cry1Ac was significantly lower in 13 field populations from northern China, where Bt cotton has been planted intensively, than in two populations from sites in northwestern China where exposure to Bt cotton has been limited. Susceptibility to Bt toxin Cry2Ab did not differ between northern and northwestern China, demonstrating that resistance to Cry1Ac did not cause cross-resistance to Cry2Ab, and implying that resistance to Cry1Ac in northern China is a specific adaptation caused by exposure to this toxin in Bt cotton. Despite the resistance detected in laboratory bioassays, control failures of Bt cotton have not been reported in China. This early warning may spur proactive countermeasures, including a switch to transgenic cotton producing two or more toxins distinct from Cry1A toxins.

## Introduction

The toxins produced by *Bacillus thuringiensis* (Bt) kill some major insect pests, but cause little or no harm to vertebrates and most other organisms [1]. Bt toxins have been used in sprays for decades and in transgenic plants since 1996 [2]. Transgenic corn and cotton producing Bt toxins grew on more than 50 million hectares worldwide in 2009 [3]. Benefits of Bt crops can include reduced use of conventional insecticides, regional pest suppression, increased yield, and increased profit [4]–[8]. The primary threat to the long-term efficacy of Bt toxins is evolution of resistance by pests, which entails a genetically based decrease in their susceptibility [9]–[12]. Many insects have been selected for resistance to Bt toxins in the laboratory, and some populations of at least six crop pests have evolved resistance to Bt toxins outside of the laboratory, including two species with resistance to Bt sprays and four species with resistance to Bt crops [12]–[14].

The main strategy for delaying pest resistance to Bt crops promotes survival of susceptible insects with “refuges” of host plants that do not produce Bt toxins [10], [15]. Ideally, most of the rare resistant insects emerging from Bt crops will mate with the relatively abundant susceptible insects from nearby refuges. If the dose of Bt toxin ingested by larvae is high enough to kill all or nearly all of the hybrid progeny produced by matings between susceptible and resistant insects, refuges are expected to be particularly effective for delaying evolution of resistance [10], [15]. Retrospective evaluations of global resistance monitoring data suggest that refuges have delayed pest resistance to Bt crops, especially when the plants have met the “high dose” criterion and refuges have been abundant [12], [16]. In the United States and Australia, farmers were required to plant refuges of non-Bt cotton near first-generation Bt cotton that produced Bt toxin Cry1Ac [12], [17]. In both of these countries, Bt cotton producing only Cry1Ac is no longer grown and has been replaced largely by Bt cotton that produces two toxins, primarily Cry1Ac and Cry2Ab [12], [17].

In China, Bt cotton producing Cry1Ac was commercialized 1997 and has been effective against the cotton bollworm, *Helicoverpa armigera*, a serious pest of many crops [8], [18]. However, the concentration of Cry1Ac declines as plants age, allowing about 5 to 20% survival of susceptible larvae toward the end of the growing season [19]. Thus, a high dose of Cry1Ac is not maintained [19], which increases the risk of resistance [10], [15]. In addition, unlike the situation in the United States and Australia, refuges of non-Bt cotton have not been required in China and Bt cotton producing Cry1Ac has not been replaced by two-toxin cotton [8], [19]. The lack of a requirement for non-Bt cotton refuges in China is based on the idea that the abundant non-Bt host plants of *H. armigera* other than cotton provide sufficient refuges to delay resistance [18]–[20]. Several monitoring studies have evaluated the success of the so-called “natural” refuge approach in China. While most previous reports have emphasized sustained susceptibility [18], [21]–[23], some data from populations sampled as recently as 2009 suggest that susceptibility to Cry1Ac may have decreased in certain limited areas [24]–[27].

Here we compared susceptibility of *H. armigera* to Cry1Ac and Cry2Ab in 2010 between 13 populations from five provinces of northern China, where Bt cotton has been planted intensively, with two populations from the Xinjiang Uygur Autonomous Region (Xinjiang) of northwestern China, where Bt cotton has not been planted intensively. The area sometimes referred to as northern China, which includes the Changjiang River Valley and the Yellow River Valley, accounts for most of China's cotton [8], [28]. In six of the provinces of northern China considered together, the percentage of cotton planted to Bt cotton increased from 11% in 1998 to 50% in 2000 and 91% in 2004, with 100% Bt cotton in some provinces by 2004 [8]. In contrast, Bt cotton has not been planted intensively in most areas of northwestern China, which accounts for about a third of China's cotton production [26]. A 2009 survey of eight locations in northwestern China where Bt cotton had not been planted showed no significant variation in susceptibility to Cry1Ac [29]. We collected and tested *H. armigera* from two sites in northwestern China in 2010: Shawan, where no Bt cotton has been planted; and Shache, where Bt cotton was first planted in 2002 and the mean percentage of cotton planted to Bt cotton from 2002 to 2009 was 5.6% (range = 0 to 11%) [26]. We used the two field populations from northwestern China and a susceptible laboratory population as susceptible standards for comparison with 13 field populations from northern China. The results show that populations of *H. armigera* from northern China have evolved resistance to Cry1Ac but not to Cry2Ab.

## Results

### Resistance to Cry1Ac

**Figure 1:**
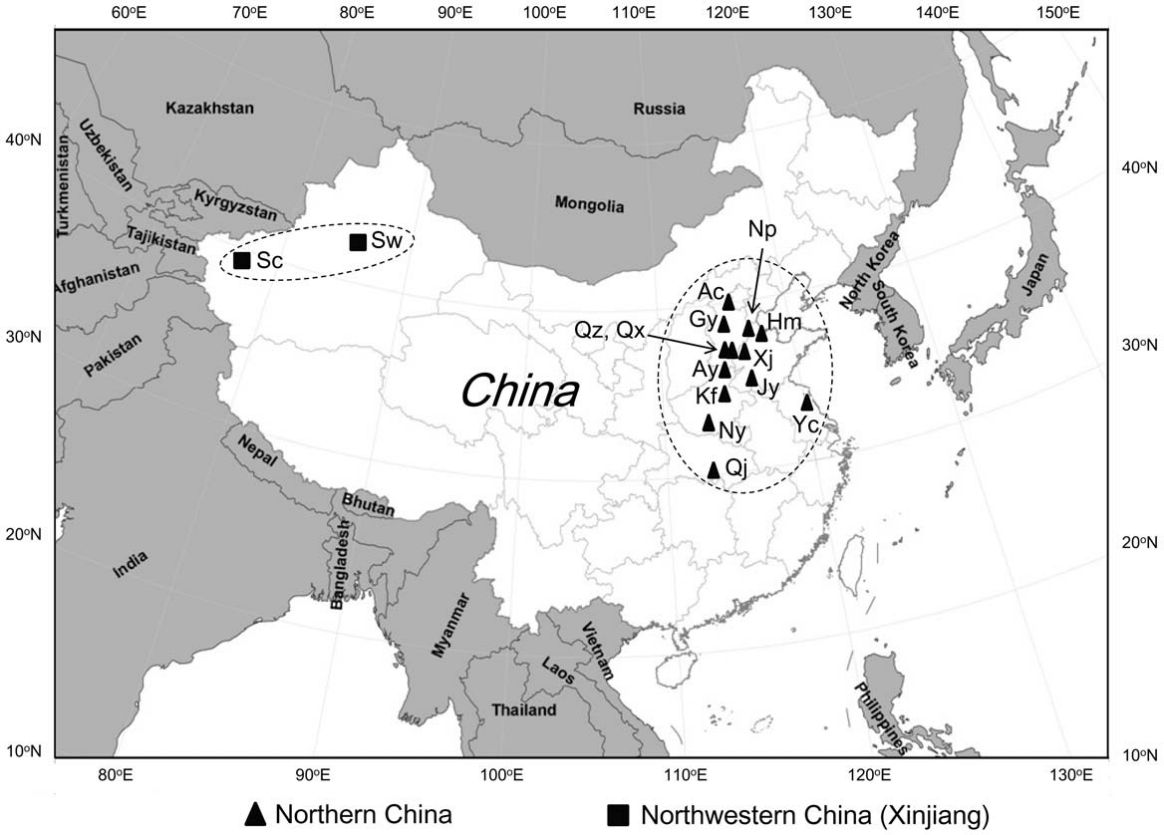
Sampling locations of *H. armigera* field populations from China. Northern China: Ac = Anci, Ay = Anyang, Gy = Gaoyang, Hm = Huimin, Jy = Juye, Kf = Kaifeng, Np = Nanpi, Ny = Nanyang, Qj = Qianjiang, Qx = Qiuxian, Qz = Quzhou, Xj = Xiajin, Yc = Yancheng. Northwestern China: Sc = Shache, Sw = Shawan.

**Figure 2:**
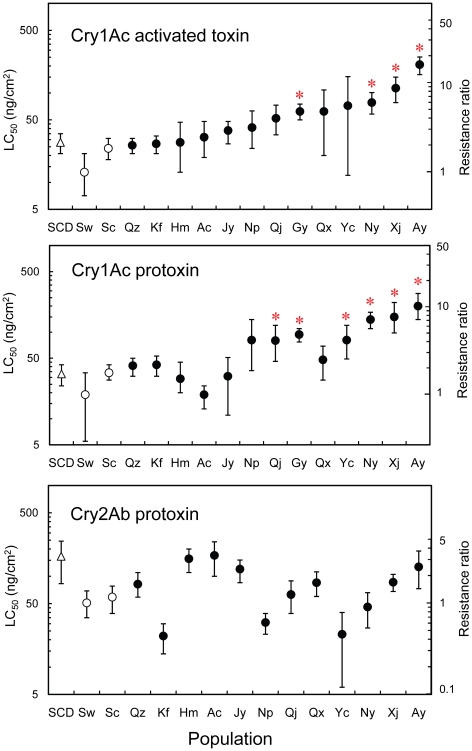
Responses to Cry1Ac activated toxin, Cry1Ac protoxin and Cry2Ab protoxin by *H. armigera* field populations sampled in 2010 from northern China (•) and northwestern China (○); and by SCD (△), a susceptible laboratory strain. LC_50_: concentration killing 50% of larvae tested with 95% fiducial limits. Resistance ratio: LC_50_ divided by the LC_50_ of the susceptible Shawan population. Asterisks indicate LC_50_ values significantly greater than the LC_50_ values of all three susceptible populations (SCD, Sc, and Sw).

Analyses of three sets of parameters from laboratory bioassays show significant resistance to Cry1Ac in populations of *H. armigera* from northern China, where Bt cotton producing Cry1Ac was planted intensively, compared with populations from two sites in northwestern China where Bt cotton was not planted intensively (Figs. 1 and 2, Tables 1, S1 and S2). The three parameter sets are: the concentration of Cry1Ac activated toxin killing 50% of larvae (LC_50_), the LC_50_ of Cry1Ac protoxin, and survival at a diagnostic concentration of Cry1Ac activated toxin. In the results of our experiments summarized below, all units for concentration are ng Bt toxin or ng Bt protoxin per cm^2^ diet.

**Table 1:** Survival at a diagnostic concentration of Cry1Ac activated toxin of *H. armigera* populations sampled in 2010 from northern China (N) and northwestern China (NW).

Location	Province	Region	Number collected	Diagnostic concentration	
				n^a^	Survival (%)
SCD^b^				168	0.0
Shawan (Sw)	Xinjiang	NW	134	1008	0.0
Shache (Sc)	Xinjiang	NW	104	288	0.0
Quzhou (Qz)	Hebei	N (YR)^c^	100	768	0.0
Kaifeng (Kf)	Henan	N (YR)	125	48	0.0
Huimin (Hm)	Shandong	N (YR)	183	1008	0.8
Anci (Ac)	Hebei	N (YR)	136	1248	1.6
Juye (Jy)	Shandong	N (YR)	117	648	0.0
Nanpi (Np)	Hebei	N (YR)	237	1848	2.2
Qianjiang (Qj)	Hubei	N (CR)^d^	457	528	0.0
Gaoyang (Gy)	Hebei	N (YR)	87	48	0.0
Qiuxian (Qx)	Hebei	N (YR)	290	888	0.2
Yancheng (Yc)	Jiangsu	N (CR)	352	1008	0.4
Nanyang (Ny)	Henan	N (CR)	150	648	1.7
Xiajin (Xj)	Shandong	N (YR)	168	1128	2.5
Anyang (Ay)	Henan	N (YR)	300	1248	2.6

a Number of F_1_ larvae tested at the diagnostic concentration (1000 ng/cm^2^).

b Susceptible strain from Cote D'Ivoire (see Methods).

c Northern China (Yellow River Valley).

d Northern China (Changjiang River Valley).

#### LC_50_ values for Cry1Ac activated toxin and protoxin

For Cry1Ac activated toxin, the median LC_50_ was 2.8 times higher for the 13 populations from northern China (52) compared with the median for the two populations from northwestern China (18.5) (Mann-Whitney U-test, U = 26, 1-tailed P = 0.0095) (Fig. 2, Table S1). The Shawan population from northwestern China, which had no exposure to Bt cotton, had the lowest LC_50_ (13) (Fig. 2, Table S1). We calculated the resistance ratio (RR) as the LC_50_ for a population divided by the LC_50_ of Shawan. For the 14 field populations other than Shawan, the RR ranged from 1.8 for the Shache population from northwestern China, which had limited exposure to Bt cotton, to 16 for the Anyang population from northern China (Fig. 2, Table S1).

We used the conservative criterion of non-overlap of 95% fiducial limits to assess differences in LC_50_ for pairwise comparisons between populations. By this criterion, the LC_50_ values did not differ significantly between Shawan and Shache from northwestern China, or between either of the two field populations from northwestern China and the susceptible SCD laboratory strain, which had no exposure to Bt toxins (Fig. 2, Table S1). However, the LC_50_ value of Cry1Ac activated toxin was significantly greater for 4 of the 13 populations from northern China (Gaoyang, Nanyang, Xiajin, and Anyang) than for each of the three susceptible populations (Shawan, Shache, and SCD; Fig. 2, Table S1).

Like the results for Cry1Ac activated toxin summarized above, the data for Cry1Ac protoxin indicate resistance of populations from northern China relative to those from northwestern China. For Cry1Ac protoxin, the median LC_50_ was 3.0 times higher for the 13 populations from northern China (80) compared with the median for the two populations from northwestern China (26.5) (Mann-Whitney U-test, U = 22.5, 1-tailed P = 0.057) (Fig. 2, Table S2). As with Cry1Ac activated toxin, Shawan had the lowest LC_50_ of Cry1Ac protoxin. For the 14 field populations other than Shawan, the resistance ratio for Cry1Ac protoxin ranged from 1.0 for Anci from northern China to 10 for Anyang from northern China (Fig. 2, Table S2). The LC_50_ value of Cry1Ac protoxin was significantly greater for 6 of the 13 populations from northern China (Qianjang, Gaoyang, Yancheng, Nanyang, Xiajin, and Anyang) than for each of the three susceptible populations (Shawan, Shache, and SCD) (Fig. 2, Table S2). Across the 15 field populations tested, the LC_50_ values for Cry1Ac activated toxin and Cry1Ac protoxin were positively correlated (Spearman's r_s_ = 0.86, df = 13, one-tailed P = 0.000019).

#### Survival at a diagnostic concentration of Cry1Ac

We used a high diagnostic concentration (1000 ng/cm^2^), which is 5.3 times the LC_99_ (190 ng/cm^2^) and slightly higher than the LC_99.99_ of the susceptible Shawan population. We chose this concentration because it was likely to kill virtually all susceptible larvae and thus provide a conservative method for detecting resistance. Also, we used this concentration previously to test the Anyang population in 2005 [30].

Survival at the diagnostic concentration was significantly higher for the 13 populations from northern China pooled (1.3%, 147 of 11,064) than for the two populations from northwestern China pooled (0%, 0 of 1296) (Table 1, Chi-squared = 18.5, df = 1, one-tailed P<0.0001). Collectively, the three susceptible populations (Shawan, Shache and SCD) had no survival at the diagnostic concentration (0 of 1464). The Anyang population, which had the highest LC_50_ values for Cry1Ac activated toxin and Cry1Ac protoxin, also had the highest survival at the diagnostic concentration (2.6%, 33 of 1248). In addition, survival at the diagnostic concentration for Anyang in 2010 was more than double the survival for Anyang in 2005 [30] (1.2%, 123 of 9984) (Chi-squared = 16.1, df = 1, one-tailed P<0.0001). Across the 15 field populations tested, survival at the diagnostic concentration and the LC_50_ of Cry1Ac activated toxin were positively correlated (Spearman's r_s_ = 0.66, df = 13, one-tailed P = 0.004).

### Susceptibility to Cry2Ab

In contrast to the results for Cry1Ac described above, the data for Cry2Ab show no significant resistance to this toxin overall in northern China relative to northwestern China. The median LC_50_ of Cry2Ab protoxin (ng/cm^2^) was not significantly higher for the 13 populations from northern China (83.5) compared with the two populations from northwestern China (55) (Mann-Whitney U-test, U = 16, 1-tailed P = 0.27) (Fig. 2, Table S3). Also, unlike the results with Cry1Ac, the lowest LC_50_ for Cry2Ab was for the Kaifeng population from northern China, rather than for the Shawan population (Fig. 2, Table S3). Indeed, the LC_50_ of Cry2Ab was significantly lower for Kaifeng than for Shawan (Fig. 2, Table S3). For Cry2Ab, none of the 13 field populations from northern China had a significantly higher LC_50_ value than each of the three susceptible populations (Shawan, Shache, and SCD) (Fig. 2, Table S3). In addition, the LC_50_ of Cry2Ab was essentially identical for the susceptible laboratory strain (SCD) and Anci, the least susceptible field population (Fig. 2, Table S3).

Across the 14 field populations tested with Cry2Ab, the LC_50_ of Cry2Ab was not significantly correlated with either the LC_50_ of Cry1Ac activated toxin or Cry1Ac protoxin (Spearman's r_s_ = 0.13 for activated toxin and −0.25 for Cry1Ac protoxin, df = 12, P>0.3 in both cases). The lack of a significant positive correlation between responses to Cry2Ab and Cry1Ac indicates that resistance to Cry1Ac did not cause cross-resistance to Cry2Ab.

## Discussion

The results reported here from three different sets of bioassay parameters show that susceptibility to Bt toxin Cry1Ac was significantly lower in 13 populations from northern China than in two populations from northwestern China. The simplest explanation for these data is that intensive planting of Bt cotton producing Cry1Ac selected for resistance to Cry1Ac in northern China, whereas limited planting of Bt cotton caused little or no selection for resistance in the two populations from northwestern China.

An alternative hypothesis is that the difference between northern and northwestern China reflects natural geographic variation in susceptibility to Cry1Ac. This hypothesis is refuted by baseline data from 1994 to 1997, before Bt cotton was planted widely in China, showing that susceptibility to Cry1Ac was not lower in northern China than in northwestern China [31]. In particular, our analysis of the baseline data (units are micrograms Cry1Ac per ml diet) shows no significant difference in median LC_50_ between 14 populations from northern China (1.2) and five populations from northwestern China (0.74) (Mann-Whitney U-test, U = 47, one-tailed P = 0.15). Unexpectedly, the baseline data show that the median concentration of Cry1Ac inhibiting development to third instar in 50% of larvae (IC_50_) was significantly lower for northern China (0.024) than for northwestern China (0.049) (Mann-Whitney U-test, U = 63, two-tailed P = 0.00036). This difference is in the opposite direction predicted by the natural variation hypothesis and thus cannot account for the decreased susceptibility to Cry1Ac in northern China relative to northwestern China detected in our 2010 monitoring.

A second line of evidence supporting the conclusion that intensive planting of Bt cotton producing Cry1Ac in northern China caused resistance is that susceptibility to Cry1Ac was significantly lower in northern China than in northwestern China in 2010, whereas no difference in susceptibility to Cry2Ab was detected in 2010 between northern China and northwestern China. These results imply that the decreased susceptibility to Cry1Ac is a specific adaptation to Bt cotton producing Cry1Ac, rather than a general difference between regions in susceptibility to Bt toxins.

A third line of evidence supporting the conclusion that intensive planting of Bt cotton producing Cry1Ac in northern China caused resistance is the significant increase in survival at the diagnostic concentration detected for Anyang in 2010 (2.6%) versus 2005 (1.2%) [30]. Among the 15 populations tested here, Anyang was the most resistant to Cry1Ac based on LC_50_ values and survival at the diagnostic concentration (Fig. 2, Tables 1, S1 and S2). Bt cotton has been planted in Anyang since 1997 and it first exceeded 90% of the total cotton area in 2001 [30].

Although the data reported here may constitute the strongest and most widespread evidence of field-evolved resistance to Cry1Ac in China, they are not the first. For example, based on F_2_ screens, Liu et al. [27] reported that the frequency of alleles conferring resistance to Cry1Ac rose 12-fold from 1999 to 2007 in the Qiuxian area of the province of Hebei in northern China. By itself, this increased frequency over time was considered ambiguous evidence of resistance because the tests in 1999 used Bt cotton plants, while tests in 2007 used Bt cotton leaves [12]. Liu et al. [27] also reported that the Qiuxian population sampled in 2007 had a Cry1Ac resistance ratio of 11, but this was based on comparison with a susceptible lab strain that was tested in a separate study.

From our bioassay results, the resistance ratios for the Qiuxian population sampled in 2010 relative to the susceptible Shawan population are 4.8 for Cry1Ac activated toxin and 2.6 for Cry1Ac protoxin (Fig. 2, Tables S1 and S2). Our 2010 results for Qiuxian also show a 0.2% survival at the diagnostic concentration of Cry1Ac (Table 1). Our results do not show significant increases in the LC_50_ of Cry1Ac or in the percentage of survivors at the diagnostic concentration for Qiuxian compared with Shawan. Nonetheless, including the previously reported data from 2007 [27] and our data from 2010 (Fig. 2, Tables S1 and S2), all evidence from Qiuxian based on five different parameters is consistent with the hypothesis of field-evolved resistance (resistance allele frequency and LC_50_ in 2007, two LC_50_ values in 2010, and survival at a diagnostic concentration in 2010; sign test, one-tailed P = 0.031).

In addition, while our main focus here is the contrast between northern and northwestern China, some recent evidence suggests that variation in susceptibility to Cry1Ac is associated with variation in the intensity of Bt cotton planting within each of these regions. Within northern China, Bt cotton planting is more intensive in Xiajin than in Anci [24], [25]. From 1998 to 2008, the mean percentage of total *H. armigera* host plant area accounted for by Bt cotton was 8.5 times higher in Xiajin (62%) than in Anci (7.3%) [24], [25]. Based on survival and mean relative average developmental rating (RADR), susceptibility to Cry1Ac was significantly lower for Xiajin than Anci in 2008 and 2009, which reflects the higher Bt cotton planting intensity in Xiajin [24], [25], [32]. Consistent with this conclusion, our results from 2010 show that susceptibility to Cry1Ac was significantly lower for Xiajin than Anci, based on the LC_50_ values for both Cry1Ac activated toxin and protoxin (Fig. 2, Tables S1 and S2).

While the intensity of Bt cotton planting generally has been lower in northwestern China than in northern China, and significant variation in the LC_50_ of Cry1Ac was not detected in a 2009 survey of eight northwestern populations from areas with no exposure to Bt cotton [29], the Korla area of northwestern China is exceptional because of its high intensity of Bt cotton planting since 2005 [26]. From 2005 to 2009, the mean percentage of total *H. armigera* host plant area accounted for by Bt cotton was 14 times higher in Korla (68%) than in Shache (5%), which is 800 km southeast of Korla [26]. From 2005 to 2009, results from experiments measuring RADR show that susceptibility to Cry1Ac decreased significantly in Korla, but not in Shache [26]. Collectively, field-evolved resistance to Cry1Ac in populations of *H. armigera* from China has been documented with monitoring data from at least seven studies based on the following comparisons: northern versus northwestern China (this study), Anci versus Xiajin in northern China [24], [ 25], [ 31, and this study], Korla versus Shache in northwestern China [26], and changes over time in northern China at both Qiuxian [27] and Anyang ([30] and this study).

We hypothesize that the resistance documented here reduces the efficacy of Cry1Ac-producing Bt cotton against *H. armigera* in the field, particularly at the end of the growing season [33]. This hypothesis is based on the finding that 5 to 20% of susceptible *H. armigera* larvae can survive on Bt cotton in China toward the end of the growing season [19], [33]. In light of only 80–95% mortality of susceptible larvae, we infer that even small decreases in susceptibility to Cry1Ac could reduce the efficacy of Bt cotton in the field. For Cry1Ac activated toxin, we found up to a 16-fold increase in the LC_50_ of Cry1Ac for a field population from northern China relative to a susceptible field population from northwestern China, and an overall tripling of the median LC_50_ of Cry1Ac activated toxin and protoxin in 13 populations from northern China relative to two populations from northwestern China (Fig. 2, Tables S1 and S2).

Additional experiments are needed to determine if the substantial decreases in susceptibility to Cry1Ac measured in lab bioassays translate to reduced efficacy in the field. Meanwhile, widespread failures of Bt cotton have not been reported in China. Two factors that could be reducing the negative impact of field-evolved resistance of *H. armigera* to Cry1Ac in northern China are the reduction in this pest's population density from 1992 to 2006 [8] and the continued application of more than 10 insecticide sprays per season on cotton [28]. Although sprays targeting *H. armigera* dropped from 1999 to 2008, the concomitant increase in sprays for mirid bugs yielded a small net increase in sprays for all insects on cotton in northern China from 1999 to 2008 [28].

The outcome of China's experiment with “natural” refuges of non-Bt host plants other than cotton is mixed. Although widespread control failures have not been reported after 14 years of commercialization of Bt cotton producing Cry1Ac, non-Bt cotton accounted for more than 20% of the total cotton planted in northern China until 2003 [8]. Thus, field-evolved resistance to Cry1Ac in northern China was detected within 8 years after Bt cotton exceeded 80% of the total area of cotton planted.

Monitoring of *H. armigera* in China has provided a warning that may be early enough to spur proactive measures to limit the consequences of resistance to Cry1Ac. The observed resistance could be countered by switching to transgenic cotton plants that produce two or more different toxins [25]. Several currently available two-toxin cultivars of cotton produce a Cry1A toxin and another toxin [12]. However, given the resistance to Cry1Ac in some field populations and the expected cross-resistance among Cry1A toxins, plants with two or more toxins other than Cry1A toxins would probably be more durable.

The results here indicating that resistance to Cry1Ac did not confer cross-resistance to Cry2Ab suggest that Cry2Ab could be useful against populations with resistance to Cry1Ac. However, our results differ from previous results based on RADR showing a genetic correlation in susceptibility between Cry1Ac and Cry2Ab both within and across populations sampled in 2008 from Anci and Xiajin [32]. Unlike previous results showing significantly decreased susceptibility to Cry2Ab in Xiajin relative to Anci in 2008 [32], we found that in 2010, the LC_50_ of Cry2Ab was slightly higher for Anci than for Xiajin (Fig. 2, Table S3).

Bt toxin Vip3Aa is especially promising for controlling populations with resistance to Cry1Ac, because susceptibility was not correlated between Cry1Ac and Vip3Aa within the Anci and Xiajin populations, and susceptibility was negatively correlated between Cry1Ac and Vip3Aa across these two populations [25]. Thus, pyramided Bt cotton producing both Vip3Aa and Cry2Ab could be particularly durable against *H. armigera*. Moreover, integration of Bt cotton with several other control tactics could provide a more sustainable pest management system [7].

## Materials and Methods

### Insect rearing and strains

We reared larvae of *H. armigera* on an artificial diet based on wheat germ and soybean powder at 27±1°C with a 16∶8 (L∶D) photoperiod. Adults were held under the same temperature and light conditions at 60–70% RH and supplied with a 10% sugar solution.

Insects were collected during June to August of 2010 from 13 sites in northern China and two sites in northwestern China (Table 1, Fig. 1). Bt cotton was the predominant host plant at all collection sites except Shawan, where no Bt cotton has been grown and non-Bt cotton was the predominant host plant. We collected male and female moths by light trap at 13 sites and eggs on Bt cotton plants at two sites in northern China (Yancheng and Qianjiang). Insects from the collected eggs were reared to adults in the laboratory on diet. We tested the F_1_ progeny from all 15 sites with bioassays as described below.

We sampled from two sites in northwestern China where Bt cotton had not been planted intensively to provide susceptible field populations for comparison with potentially resistant field populations from northern China, where Bt cotton had been planted intensively. We did not sample more sites throughout northwestern China because our goal was to use the two northwestern populations as standards for comparison, not to assess variation in northwestern China, which has been reported previously [26], [29]. As another standard for comparison, we also tested the susceptible SCD strain. The SCD strain was started with insects from the Cote D'Ivoire (Ivory Coast), Africa over 30 years ago and has been maintained in the laboratory with no outcrossing and no exposure to insecticides or Bt toxins [34].

### Bt toxins

Cry1Ac protoxin was produced from the HD73 strain of *Bacillus thuringiensis* subsp. *kurstaki*. Activated Cry1A toxin was prepared by incubating with 20∶1(w/w) of protoxin∶TPCK-treated bovine trypsin (Sigma, T-8642). Cry2Ab protoxin was provided by the Institute of Plant Protection, Chinese Academy of Agricultural Sciences (CAAS), China.

### Bioassays

We used diet surface overlay bioassays. Toxin stock suspensions were diluted with a 0.01 M, pH 7.4, phosphate buffer solution (PBS). Liquid artificial diet (900 µl) was dispensed into each well of a 24-well plate. After the diet cooled and solidified, 100 µl of PBS containing the desired concentration of Bt toxin was applied evenly to the diet surface in each well and allowed to air dry, and a single larva was placed in each well. At the end of the bioassay, we scored larvae as dead if they died or if they weighed less than 5 mg.

For Cry1Ac, we used second instars that were starved for 4 h and we recorded mortality at 5 days. This method is identical to our previous method with Cry1Ac [30], [35], except that here the diet was dispensed into wells as a liquid, which is more efficient than our previous method of inserting a disc of diet into each well [30]. Calibration tests with the SCD strain showed that compared with the old method [30], the LC_50_ of Cry1Ac was 3- to 5-fold lower with the new method. For any given concentration, survival was lower for the new method than the old method. This means that any increase in survival found with the new method compared with previous results from the old method is conservative because it would tend to underestimate the increase in survival.

For Cry2Ab, we used unfed neonates (<24 h old) and recorded mortality after 7 days. The method for Cry2Ab required less toxin than the method for Cry1Ac, and followed the method established in Australia for testing Cry2Ab against *H. armigera* [36]. We were not able to test the Gaoyang population with Cry2Ab.

For all concentrations other than the diagnostic concentration of Cry1Ac activated toxin (1000 ng/cm^2^), we tested 48 larvae for each toxin concentration, including a control with PBS and no toxin. For the diagnostic concentration, the number of larvae tested per population ranged from 48 to 1848 (Table 1). All tests were done at 26±1°C, with a 16∶8 L∶D photoperiod and 60% RH. Control mortality was consistently low across all populations tested (mean = 2.5%, range = 0 to 6.2%).

### Data analysis

We used the PoloPlus program [37] to conduct probit analysis of the concentration-mortality data to estimate the concentration killing 50% of larvae tested (LC_50_), the 95% fiducial limits of the LC_50_, the slope of the concentration-mortality line and the standard error of the slope. We considered two LC_50_ values significantly different only if their 95% fiducial limits did not overlap, which is a conservative criterion [38], [39]. We calculated the resistance ratio (RR) as the LC_50_ of a population divided by the LC_50_ of the Shawan field population, which had not been exposed to Bt cotton.

To test the hypothesis that the LC_50_ values were higher for northern China (which had a history of intense planting of Bt cotton) than northwestern China (which had a history of much less planting of Bt cotton), we used the Mann-Whitney U-test. We used Spearman's rank correlation to test the hypothesis that positive correlations occurred across populations between each of the following four pairs of parameters: LC_50_ of Cry1Ac activated toxin and Cry1Ac protoxin, LC_50_ and survival at the diagnostic concentration of Cry1Ac activated toxin, LC_50_ of Cry1Ac activated toxin and Cry2Ab protoxin, LC_50_ of Cry1Ac activated toxin and Cry2Ab protoxin. We used a Chi-squared test to determine if the frequency of survivors at the diagnostic concentration was higher in northern China than in northwestern China, and higher in 2010 (based on data here) than in 2005 (data from Yang et al. [30]) for Anyang. Because each hypothesis we tested is one-sided, we used one-tailed probability values.

## Supporting Information

Table S1 (PDF)

Table S2 (PDF)

Table S3 (PDF)
