# Supplementary material for: Early Warning of Cotton Bollworm Resistance Associated with Intensive Planting of Bt Cotton in China
Source: PLoS One. 2011 Aug 9;6(8):e22874. doi: 10.1371/journal.pone.0022874 (PMC3153483; doi:10.1371/journal.pone.0022874)
Supplement: Table S1 — (PDF) [file pone.0022874.s001.pdf]

Table S1. Responses to Cry1Ac activated toxin by *H. armigera* field populations sampled in 2010 from northern China (N) and northwestern China (NW)

Population	Region	n	Slope±SE	LC_{50}[a] (ng/cm^2)	95% fiducial limits of LC_{50}	RR[b]
SCD[c]		408	2.2±0.2	28	21 - 35	2.2
Shawan (Sw)	NW	1200	2.1±0.3	13	7.1 - 21	1.0
Shache (Sc)	NW	528	1.9±0.2	24	18 - 31	1.8
Quzhou (Qz)	N	960	2.6±0.3	26	21 - 31	2.0
Kaifeng (Kf)	N	288	2.9±0.4	27	21 - 33	2.1
Huimin (Hm)	N	1248	1.6±0.1	28	13 - 47	2.2
Anci (Ac)	N	1536	1.5±0.1	32	19 - 48	2.5
Juye (Jy)	N	840	2.2±0.2	38	27 - 48	2.9
Nanpi (Np)	N	2040	1.5±0.1	41	24 - 63	3.2
Qianjiang (Qj)	N	768	2.1±0.2	52	34 - 73	4.0
Gaoyang (Gy)	N	336	2.0±0.2	62	50 - 75	4.8
Qiuxian (Qx)	N	1224	2.1±0.3	62	20 - 108	4.8
Yancheng (Yc)	N	1248	2.1±0.2	72	12 - 152	5.5
Nanyang (Ny)	N	936	1.9±0.1	78	58- 101	6.0
Xiajin (Xj)	N	1416	2.0±0.1	113	78 - 150	8.7
Anyang (Ay)	N	1584	2.8±0.1	207	160 - 252	16

[a] Concentration killing 50% of larvae tested

[b] Resistance ratio; LC_{50} of a population divided by the LC_{50} of the susceptible Shawan population

[c] Susceptible laboratory strain
