# Supplementary material for: Early Warning of Cotton Bollworm Resistance Associated with Intensive Planting of Bt Cotton in China
Source: PLoS One. 2011 Aug 9;6(8):e22874. doi: 10.1371/journal.pone.0022874 (PMC3153483; doi:10.1371/journal.pone.0022874)
Supplement: Table S2 — (PDF) [file pone.0022874.s002.pdf]

Table S2. Responses to Cry1Ac protoxin by *H. armigera* field populations sampled in 2010 from northern China (N) and northwestern China (NW)

Population	Region	n	Slope±SE	LC_{50}[a] (ng/cm^2)	95% fiducial limits of LC_{50}	RR[b]
SCD[c]		240	2.6 ± 0.4	33	24 - 42	1.7
Shawan (Sw)	NW	240	2.1 ± 0.3	19	5.5 - 34	1.0
Shache (Sc)	NW	288	2.1 ± 0.2	34	28 - 42	1.8
Quzhou (Qz)	N	240	2.6 ± 0.4	41	31 - 50	2.2
Kaifeng (Kf)	N	240	2.3 ± 0.3	42	31 - 53	2.2
Huimin (Hm)	N	240	2.0 ± 0.3	29	20 - 45	1.5
Anci (Ac)	N	288	2.1 ± 0.3	19	13 - 24	1.0
Juye (Jy)	N	288	1.6 ± 0.2	31	11 - 51	1.6
Nanpi (Np)	N	288	1.4 ± 0.2	81	36 - 140	4.3
Qianjiang (Qj)	N	240	2.3 ± 0.3	80	46-120	4.2
Gaoyang (Gy)	N	240	2.4 ± 0.3	94	77 - 110	4.9
Qiuxian (Qx)	N	288	1.6 ± 0.2	48	28 - 69	2.5
Yancheng (Yc)	N	192	1.4 ± 0.2	81	49 - 120	4.3
Nanyang (Ny)	N	192	3.5 ± 0.6	140	110 - 170	7.5
Xiajin (Xj)	N	288	2.2 ± 0.2	150	98 - 220	7.7
Anyang (Ay)	N	288	2.6 ± 0.2	200	140 - 280	10

[a] Concentration killing 50% of larvae tested

[b] Resistance ratio; LC_{50} of a population divided by the LC_{50} of the susceptible Shawan population

[c] Susceptible laboratory strain
