# Supplementary material for: Early Warning of Cotton Bollworm Resistance Associated with Intensive Planting of Bt Cotton in China
Source: PLoS One. 2011 Aug 9;6(8):e22874. doi: 10.1371/journal.pone.0022874 (PMC3153483; doi:10.1371/journal.pone.0022874)
Supplement: Table S3 — (PDF) [file pone.0022874.s003.pdf]

Table S3. Responses to Cry2Ab protoxin by *H. armigera* field populations sampled in 2010 from northern China (N) and northwestern China (NW)

Population	Region	n	Slope±SE	LC_{50}[a] (ng/cm^2)	95% fiducial limits of LC_{50}	RR[b]
SCD[c]		288	2.3±0.3	170	83-240	3.3
Shawan (Sw)	NW	336	1.7 ± 0.2	51	35 - 69	1.0
Shache (Sc)	NW	288	2.1 ± 0.3	59	39 - 78	1.2
Quzhou (Qz)	N	288	2.1 ± 0.3	82	59 - 110	1.6
Kaifeng (Kf)	N	336	1.6 ± 0.2	22	14 - 30	0.43
Huimin (Hm)	N	336	2.0 ± 0.2	156	110 - 200	3.1
Anci (Ac)	N	384	1.7 ± 0.2	170	100 - 240	3.3
Juye (Jy)	N	384	2.2 ± 0.3	120	85 - 151	2.3
Nanpi (Np)	N	288	2.3 ± 0.3	31	23 - 39	0.61
Qianjiang (Qj)	N	288	2.0 ± 0.2	63	39 - 89	1.2
Qiuxian (Qx)	N	336	1.7 ± 0.2	85	60 - 110	1.7
Yancheng (Yc)	N	240	2.1 ± 0.3	23	6.0 - 40	0.45
Nanyang (Ny)	N	336	1.4 ± 0.2	46	27 - 66	0.9
Xiajin (Xj)	N	288	2.5 ± 0.3	86	68 - 100	1.7
Anyang (Ay)	N	336	1.9 ± 0.2	130	73 - 190	2.5

[a] Concentration killing 50% of larvae tested

[b] Resistance ratio; LC_{50} of a population divided by the LC_{50} of the susceptible Shawan population

[c] Susceptible laboratory strain
